# Methanolic Extract of *Distemonanthus benthamianus* (Caesalpiniaceae) Stem Bark Suppresses Ethanol/Indomethacin-Induced Chronic Gastric Injury in Rats

**DOI:** 10.1155/2020/8180323

**Published:** 2020-11-28

**Authors:** Vanessa Mba Matah Marte, Gilbert Ateufack, Marius Mbiantcha, Albert Donatien Atsamo, Carine Flore Adjouzem, Stéphanie Flore Djuichou Nguemnang, Eric Gonzal Tsafack, William Yousseu Nana, Yacine Karelle Madjo Kouam, Elvira Ngoufack Azanze

**Affiliations:** ^1^Laboratory of Animal Physiology and Phytopharmacology, Faculty of Science, University of Dschang, Cameroon; ^2^Laboratory of Animal Physiology, Faculty of Science, University of Yaounde I, P.O. Box 812, Yaoundé, Cameroon

## Abstract

*Distemonanthus benthamianus* (Caesalpiniaceae) is a plant from the Cameroon pharmacopoeia very widely used in the treatment of many pathologies among which are gastrointestinal disorders. The main purpose of this study was to assess the healing properties of gastric ulcer from the methanolic extract of *Distemonanthus benthamianus* and its mechanisms of action. The healing properties of gastric ulcers (chronic ulcer model induced by ethanol and indomethacin) were evaluated *in vivo* in adult male rats, while the mechanisms of action were evaluated *in vitro* by anti-inflammatory assay (protein denaturation, cyclooxygenase, and lipoxygenase assays) and immunomodulatory assay (ROS production (using technical chemiluminescence), cytokine (TNF-*α*, IL-1*β*, IL-6) production (using ELISA), proliferation of T cells (using liquid scintillation counter), and cytotoxicity (using MTT assay)). The methanolic extract of *Distemonanthus benthamianus* inhibited protein denaturation (75.63%) and the activities of cyclooxygenase (78.92%) and 5-lipoxygenase (81.54%). The extract also significantly (*p* < 0.001) inhibited intracellular and extracellular ROS production and T cell proliferation and reduced significantly (*p* < 0.01, *p* < 0.001) TNF-*α*, IL-1*β*, IL-6, and PGE2 production. At all doses (125, 250, and 500 mg/kg), the extract significantly reduces the ulceration index and the area of ulceration and significantly increases the mass of gastric mucus. In addition, the extract significantly decreases the level of MDA, significantly increases the activities of catalase and glutathione, and then improves the hematological parameters in sick animals. Histological micrographs show that in the presence of the extract, there is advanced reepithelialization with recovery of the ulcerated epithelium. Thus, the extract of *Distemonanthus benthamianus* has healing properties against gastric ulcers which are associated with its anti-inflammatory, immunomodulatory, and antioxidant effects.

## 1. Introduction

Caesalpiniaceae represent a plant family made up of subtropical and tropical shrubs and trees with more than 150 genera and around 2200 species. The leaves have the characteristics of being stipulated, alternate, pinnate, bipinnate, or simple with a petiole very often enlarged at the base. The flowers of this plant family, zygomorphic and strongly perigynous, appear in spikes, in clusters, or in cymes. Their fruits are generally legumes [1]. Several species of Caesalpinioideae are highly sought after because of their ornamental characteristics. In addition, several of these species produce numerous resins, very precious wood, and above all medicinal substances [2]. *Distemonanthus benthamianus* (*D. benthamianus*) is a species belonging to the large family of Caesalpiniaceae and which is very widespread in Africa where it is widely used for its many therapeutic virtues.

*D. benthamianus*, still known as Movingui in Gabon, Barre in Ivory Coast, Bonsamdua in Ghana, Eyen in Cameroon, and Ayan in Nigeria, is one of the largest trees widespread in Africa, with a height of 90 to 125 m and evergreen. On the ethnopharmacological level, the barks, leaves, and roots of this plant are used to treat many pathologies among which are nervous disorders, constipation, digestive disorders, and dropsy. A decoction of the mixture of leaves and bark is used to treat many bacterial infections [3]. In the western region of Nigeria, the Yaouba people use the stem and roots as a chewing stick for oral hygiene [4]. Several studies have shown that extracts from *D. benthamianus* are rich in alkaloids, flavonoids, polyphenols, saponins, sterols, tannins, and triterpenes [3, 5]. The work of Nguelefack et al. [3] has shown that *D. benthamianus* has antibacterial properties in vitro against Staphylococcus aureus and Streptococcus agalactiae, while the work of Yousseu et al. [5] has shown the antidiarrheal properties of the aqueous and methanolic extracts of this plant. Other work has shown that extracts of *D. benthamianus* have spasmolytic and muscle relaxant properties by blocking voltage-gated calcium channels and by inhibition of muscarinic receptors [6]. In addition, an analysis by HPLC of the methanolic extract of *D. benthamianus* showed the presence of gallic acid, which is known for its numerous pharmacological properties, in particular on pathologies of the gastrointestinal tract [7]. The aqueous and methanolic extracts of this plant considerably reduce the serum levels of proinflammatory cytokines (TNF-*α* and IL-1*β*) and facilitate the reconstitution of tissues in the ileum and the colon mucosa [7]; this could justify a potential anti-inflammatory or healing effect of extracts from this plant.

Peptic ulcer is a more or less serious and frequent injury (stomach and duodenum) resulting from a significant imbalance between the endogenous aggressive factors of the wall and the protective factors [8]. Its incidence is greatly increased by factors such as smoking, Helicobacter pylori, alcohol consumption, the use of nonsteroidal anti-inflammatory drugs (NSAIDs), and stress [9], since these factors promote the development and maintenance of lesions of the gastric mucosa with the consequence of the persistence of ulcers with triggering of an inflammatory process. Consumption of alcohol and/or NSAIDs results in the development of ulceration, erosion of the gastric mucosa, perforation, and bleeding [10, 11]; these effects are consecutive to the excessive production of gastric acid, proinflammatory cytokines (TNF-*α* and IL-1*β*), and reactive oxygen species (ROS), to the increase in lipid peroxidation, to infiltration of neutrophils, and even to apoptosis [12–14]. The increase in all these factors leads to the development and maintenance of an inflammatory process which is characterized by the increase in the activities of cyclooxygenase-2 (COX-2) and 5-lipoxygenase (5-LOX) and an increase in the denaturation of proteins which represent the aggravating factors of inflammation [15].

Based on the fact that preliminary in vitro studies of this study have shown that the stem bark of the methanolic extract of *D. benthamianus* has the property of significantly inhibiting protein denaturation, the activities of COX and 5-LOX, the production of proinflammatory cytokines (TNF*α*, IL-1*β*, IL6), the production of PGE2, and the production of ROS as well as cell proliferation and in addition, this extract is rich in alkaloids, flavonoids, cardiac glycosides, steroids, triterpenoids, tannins, and saponins [3, 5] and has gallic acid [7], we can conclude that this plant may have healing properties of gastric ulcers. The objective of this work was to evaluate the healing properties of the methanolic extract of *D. benthamianus* on the model of chronic ulcer induced by the ethanol and/or indomethacin.

## 2. Materials and Methods

### 2.1. Plant Material and Extraction

The sample of *D. benthamianus* (leaves, barks, and flower) was collected in Souza (Littoral region, Cameroon) in November 2017 and a comparison with specimen no. 45488 HCN was authenticated at the national herbarium in Yaoundé (Cameroon). The bark of this plant was discarded, chopped, dried in the shade, and reduced to powder.

The powder (300 g) was soaked in 3 liters of methanol for 72 h. After filtration, a Büchi rotary evaporator (R-124) set at 65°C was used to concentrate the filtrate under reduced pressure. Twenty-two grams (22 g) of methanolic extract (yield 7.3% (*w*/*w*)) was obtained.

### 2.2. *In Vitro* Assay

#### 2.2.1. Reagents, Chemicals, and Equipment

Lymphocyte separation medium, luminol, indomethacin, lucigenin, and Hanks Balanced Salt Solution were obtained from MP Biomedicals Inc., Sigma, and Research Organics; ethanol, ammonium chloride of analytical grades, and dimethyl sulfoxide (DMSO) from Merck Chemicals, Darmstadt, Germany; Zymosan A as phorbol myristate acetate from Fluka; and human monocytic leukemia cells from European Collection of Cell Cultures; trypsin and casein were procured from HiMedia Lab. Ltd, Mumbai. Potassium persulfate, N-(1-naphthyl) ethylenediamine dihydrochloride, glutathione, potassium phosphate buffer, and benzene were obtained from LOBA CHEMIE Pvt. Ltd. Mumbai. RPMI 1640 medium and phytohemagglutinin were from Hi-Media. CFA was purchased from Sigma Chemical Co. (St. Louis, MO, USA), while diclofenac (Olfen-100 SR), all other chemicals and reagents were bought in a locally certified pharmacy.

#### 2.2.2. Anti-Inflammatory Assay

*(1) Inhibition of Protein Denaturation*. The extract or diclofenac sodium (1 ml) at concentrations of 100, 200, 500, and 1000 *μ*g/ml was mixed with bovine serum albumin (5%, 1 ml) and then incubated at 27°C (15 min). The negative control had only a solution of distilled water and bovine serum albumin. The tubes were placed in a water bath (70°C, 10 min) to cause denaturation of the proteins. Each tube was left for cooling, and the optical density was read at 660 nm. Each test was carried out in three repetitions [16]. The percentage inhibition was calculated using the following formula:

(1) $$\begin{array}{c} \%\text{Inhibition}=\frac{\text{Absorbance of control}-\text{Absorbance of sample}}{\text{Absorbance of control}}\times 100. \end{array}$$

*(2) Cyclooxygenase and 5-Lipoxygenase Assay*. (*1) Preparation of Lymphocyte Culture*. A culture of human peripheral lymphocytes (RPMI 1640, 20% fetal bovine serum, and antibiotics) stimulated by phytohemagglutinin was filtered (cellulose acetate of 0.2 *μ*m); after addition of fresh plasma (1 × 10^6^ cells/ml), it was incubated for 72 h. Then, 1 *μ*l of lipopolysaccharides was added to the culture which was then incubated for 24 h. The extract or ibuprofen (100 and 500 *μ*g/ml, final concentration) was then added, and a new 24-hour incubation was carried out. The tubes were then centrifuged (6000 rpm, 10 min) for isolation, the supernatant was removed, and then, the cell lysis buffer (50 *μ*l) was added, and the tubes were again centrifuged (6000 rpm, 10 min); then, the pellet was kept for anti-inflammatory tests [17].

*(2) Cyclooxygenase Assay*. The arachidonic acid was mixed with glutathione, a tripeptide; Tris-HCl buffer; and hemoglobin and then incubated (37°C, 20 min). Then, 0.2 ml of TCA (10%, 1 N HCl) and 0.2 ml of TBA were also added and the contents were passed through a boiling water bath (20 min). After cooling and centrifuging (1000 rpm) for 3 min, the COX activity was determined in the supernatant by reading the optical density at 632 nm [17].

*(3) 5-Lipoxygenase Assay*. Sodium hydroxide (0.5 N) was added to a Tween 20 bubble-free mixture, linoleic acid (70 mg), and deoxygenated water (4 ml) to give a 25 ml solution. This solution (0.5 ml) was distributed into tubes, and nitrogen gas was added to each tube which was closed and kept in the freezer. A quartz bowl (25°C) with a light path of 1 cm allowed the reaction to take place. The test tubes contained Tris buffer (2.75 ml, pH 7.4), sodium linoleate (0.2 ml), and the enzyme (50 ml). The activity of 5-LOX was determined by reading the optical density at 234 nm [17].

#### 2.2.3. Immunomodulatory Essay

*(1) Isolation of Human Polymorphoneutrophils (PMN)*. Blood (10 ml) freshly drawn from an adult and healthy person was introduced into a heparinized tube; then, the lymphocyte separation medium and Hanks Balanced Salt Solution (HBSS^--^) were added to the tube which was left to stand (about 30 min). After good separation of the solution, the supernatant was removed and introduced into tubes each containing 5 ml of lymphocyte separation medium and then centrifuged (400 × g, 20 min, room temperature). The supernatant was then removed, 1 ml of distilled water was added for exactly 1 min, and then, 1 ml of HBSS^--^ was also added. Subsequently, HBSS^--^ (5 ml) was further added to each tube which was then centrifuged at 300 × g (4°C) for 10 min. After removing the supernatant, HBSS^--^ (1 ml) was added again to each tube which was kept in ice. Both cell number and viability were determined by the trypan blue exclusion method [18]. The concentration used for each test was 1 × 10^6^ cells/ml.

The human blood samples used in this work were received from a donor following the procedure accepted by the Independent Ethics Committee, ICCBS, University of Karachi, No. ICCBS/IEC-008-BC2015/Protocol/1.0. Blood donors have been informed that it is to be used for an experimental study.

*(2) Isolation of Mouse Peritoneal Macrophages*. Three NMRI (Naval Medical Research Institute) mice weighing an average of 23 g received fetal bovine serum (1 ml, i.p.). After 72 h and sacrifice (cervical dislocation), 10 ml of RPMI (Roswell Park Memorial Institute) medium 10% was injected into the peritoneum of each animal. 2 min after injection, the RPMI medium was withdrawn using a syringe introduced into the animals' peritoneum. The liquid was transferred to tubes which were centrifuged at 400 × g (4°C) for 20 min; then, the supernatant was removed and 5 ml of incomplete RPMI was added again followed by further centrifugation (300 × g, 4°C, 10 min). After removal of the supernatant, incomplete RPMI/HBSS^--^ medium (1 ml) was added to each tube. Both cell number and viability were determined by the trypan blue exclusion method [19, 20]. The same cell concentration as the PMN was used for each test.

*(3) Chemiluminescence Assay*. The chemiluminescence test was carried out in white 96-well plates according to the modified methods of Mesaik et al. [19] and Mahomoodally et al. [20]. Thus, 3.1 to 100 *μ*g/ml of extract (25 *μ*l) or ibuprofen (25 *μ*l) and blood (25 *μ*l, diluted (1 : 50)) or PMN (25 *μ*l) or macrophages (25 *μ*l) was mixed in different wells. Control wells contained only HBSS^++^ (25 *μ*l), blood (25 *μ*l), or PMN (25 *μ*l) or macrophages (25 *μ*l). After incubation (20 min, 37°C) of the plate in the thermostated luminometer chamber, the zymosan/PMA mixture (50 *μ*l) for the extracellular ROS or the luminol/lucigenin mixture (50 *μ*l) for the intracellular ROS was added in the respective wells. The plate was directly introduced into the luminometer, and the results were obtained in relative light units (RLU) [21], and the percentage of inhibition was calculated by the following formula:

(2) $$\begin{array}{c} \text{Inhibition}\left(\%\right)=\frac{\left({\text{RLU}}_{\text{control}}-{\text{RLU}}_{\text{sample}}\right)\times 100\%}{{\text{RLU}}_{\text{control}}}. \end{array}$$

*(4) Proinflammatory Cytokine Assay*. The peritoneal macrophages of harvested mice were washed twice in PBS, adjusted to 10^6^ cells/ml, and cultured in CO_2_ (37°C, 24 h), in RPMI, and/or with lipopolysaccharides (LPS; 2 *μ*g/ml) with or without extract (2, 10, and 50 *μ*g/ml). After 24 h, the mixture was centrifuged (2500 rpm, 20 min); then TNF-*α*, IL-6, IL-1*β*, and PGE2 were determined in the supernatant using the ELISA kits (ELAB Sciences) and following the manufacturer's instructions [22].

*(5) Cell Proliferation Assay*. To evaluate the effect of the methanolic extract of *D. benthamianus* on cell proliferation, the 96-well white round-bottom plates were used and the protocol described by Mesaik et al. [19] has been used. The extract (50 *μ*l) or prednisolone (50 *μ*l) of varying concentrations of 2, 10, and 50 *μ*g/ml was mixed with RPMI (5%) in the different wells. After adding 50 *μ*l of T cells (2 × 10^6^ cells/ml), each well was stimulated by adding 50 *μ*l of phytohemagglutinin-L (PHA-L) (7.5 *μ*g/ml). The cells (50 *μ*l) and 150 *μ*l of 5% RPMI constituted the mixture of the negative control wells while the positive control wells consisted of a mixture of cells (50 *μ*l), of PHA (50 *μ*l), and of 5% RPMI (100 *μ*l). After incubation (5% CO_2_, 72 h, 37°C) of the plate, 25 *μ*l of methyl-3H-thymidine (0.5 *μ*Ci) was added to each well and the plate was incubated again for 18 h and cells were harvested using a fiberglass filter. An IS65000 liquid scintillation counter was used to determine the level of thymidine integrated into the cells. The percentage inhibition was determined using the counts per minute (CPM) of each well according to the following formula:

(3) $$\begin{array}{c} \text{I}nhibition\;activity\;\left(\%\right)=\frac{{\text{CPM}}_{\left(\text{control group}\right)-{\text{CPM}}_{\left(\text{test group}\right)}}}{{\text{CPM}}_{\left(\text{control group}\right)}}\times 100. \end{array}$$

*(6) MTT Cytotoxicity Assay*. A cell suspension (100 *μ*l, 6 × 10^4^ cells/ml) introduced into wells of a 96-well plate was incubated (5% CO_2_, 37°C) for 24 h; then, the medium was removed and then, 3.1 to 100 *μ*g/ml of extract and the complete DMEM was added to each well to give a final volume of 200 *μ*l per well. 100 *μ*l of cell and the DMEM constituted the mixture of positive control wells while the negative control wells received an additive of 2 *μ*l of Triton X-100 (0.5%). The plate was then incubated for 48 h (CO_2_, 37°C), the supernatant was removed, and then, 50 *μ*l of MTT (0.5 mg/ml) was added to each and then followed by a new incubation for 4 h. After aspiration of the MTT, 100 *μ*l of DMSO was added; then, the plate was shaken (orbital shaker) for 10 to 15 min, and the absorbance was read at 540 nm [23]. The percentage of inhibition or decrease in cell viability was obtained by the following formula:

(4) $$\begin{array}{c} \%\text{Inhibition}=100-\frac{{\text{OD}}_{\text{test group}}-{\text{OD}}_{\text{blank}}}{{\text{OD}}_{\text{control group}}-{\text{OD}}_{\text{blank}}}\times 100. \end{array}$$

### 2.3. *In Vivo* Assay

#### 2.3.1. Chemicals and Drugs

DMSO, ethanol, and indomethacin were obtained from Sigma Chemicals. Sucralfate was purchased from an accredited pharmacy (Martyrs, Bafoussam, Cameroon). All chemicals and reagents used were of analytical grade.

#### 2.3.2. Animals

Male *Wistar* rats weighing between 150 and 200 g were used. Under natural conditions, they were raised in the animal house of the Animal Biology Department of Dschang University, Cameroon. The animals were fed a standard diet and were given unlimited water. In order to minimize any nonspecific stress, the rats were acclimated for 48 h before the experiment. The experimental protocols used in this study have been approved by the laboratory committee (Research Unit in Animal Physiology and Phytopharmacology, Department of Animal Biology, Faculty of Science, University of Dschang, Cameroon), in accordance with standard ethical guidelines for the use and the care of laboratory animals, described in the European Community guidelines; EEC Directive 86/609/EEC of 24 November 1986 [24].

#### 2.3.3. Distribution and Treatment of Animals

Forty-two rats were fasted for 48 h. These animals were divided into 7 groups of 6 rats each: groups 1 and 2 composed of rats which will not receive any treatment, group 3 composed of rats which will receive DMSO 3%, group 4 which will receive sucralfate (100 mg/kg), and groups 5, 6, and 7 who will receive the methanolic extract of *D. benthamianus* at the respective doses of 125, 250, and 500 mg/kg.

#### 2.3.4. Induction of Gastric Ulcers

Chronic gastric ulcers were induced using the technique described by Wang et al. [25] with modifications. In the first 5 days, 1 ml of absolute ethanol (70%) was administered orally to animals (except group 1 rats) for the induction of gastric ulcers. On day 6 after the start of this administration, the animals in group 2 were sacrificed; then, their stomach was opened, and the ulcerated surface and the mass of the mucus were measured to confirm the chronicity of the ulcer.

From day 7, animals in groups 3 to 7 received indomethacin by intraperitoneal injection (10 mg/kg, 1 ml/200 g body weight) for 4 consecutive days; subsequently, these animals received the various oral treatments daily for 10 days.

#### 2.3.5. Macroscopic Assessment of the Ulcerated Area and Blood Parameters

At the end of the treatment, (10^th^ day), the animals were sacrificed after anesthesia performed using diazepam/ketamine, the stomach was removed and opened along the greater curvature and washed, and mucosal lesions were noted as described by Adinortey et al. [26]. The ulcerated surface, the mass of mucus, and the index of ulceration were determined. The ulcer index (UI) for each rat was recorded according to the average score for ulcer: 0: normal mucosa; 1: 1-4 small petechiae; 2: 5 or more petechiae or hemorrhagic streaks up to 4 mm long; and 3: erosion of more than 5 mm or confluent hemorrhages. Photographs of the gastric mucosa were taken. The cure rate for ulcers (UH %) was calculated by the following formula:

(5) $$\begin{array}{c} \text{UH}\left(\%\right)=\frac{{\text{UI}}_{\text{control}}-{\text{UI}}_{\text{treated}}}{{\text{UI}}_{\text{control}}}\times 100. \end{array}$$

After sacrifice, catheterization of the abdominal artery was performed to collect blood in a tube filled with anticoagulant (EDTA) for the analysis of hematological parameters (leukocytes (WBC), lymphocytes, monocytes, neutrophils, eosinophils, and basophils). Then, the stomach was removed and part was removed, ground in a buffer, and centrifuged at 3000 rpm for 15 min at 4°C; then, the supernatant of the gastric homogenates was removed to measure the oxidative stress parameters such as cellular glutathione (GSH) according to the method described by Ellman [27], superoxide dismutase (SOD) according to the method described by Misra and Fridovich [28], catalase according to the method described by Sinha [29], and malondialdehyde (MDA) according to the method described by Wilbur et al. [30].

### 2.4. Histological Examinations

The rest of the stomach was kept in 10% formalin solution, followed by tissue dehydration with alcohol and xylene. Each sample was included in paraffin, sectioned at 5 *μ*m on thin slides, stained with hematoxylin/eosin mixture, and finally observed under an optical microscope.

## 3. Statistical Analyses

One-way analysis of variance (ANOVA) was used to analyze the data, followed by a Tukey post hoc test. Statistical significance was acceptable at *p* < 0.05, and all data are plotted as mean ± SEM. The software program R.3.5.0 was used.

## 4. Results

### 4.1. *In Vitro* Assay

#### 4.1.1. Anti-Inflammatory Activities

*(1) Effect of Methanolic Extract of D. benthamianus on the Inhibition of Protein Denaturation*.  Table 1 presents the effect of the methanolic extract of *D. benthamianus* on the denaturation of proteins. It appears from this table that the extract inhibited the denaturation of proteins caused by heat. Indeed, a significant inhibition (*p* < 0.001) of 58.52%, 60.39%, 64.18%, and 75.63% is obtained with the extract at concentrations of 100, 200, 500, and 1000 *μ*g/ml, while diclofenac produced an inhibition of 74.41%, 76.72%, 81.48%, and 89.19% at the same concentrations.

*(2) Effect of Methanolic Extract of D. benthamianus Inhibiting the Activity of Cyclooxygenase*. The evaluation of the effect of the extract on the activity of cyclooxygenase determines the effect of the extract on the production of prostaglandins (Table 1). It appears from the result obtained that at concentrations of 500 and 1000 *μ*g/ml, the methanolic extract of *D. benthamianus* significantly inhibits (*p* < 0.001) the activity of cyclooxygenase by 64.31% and 78.92%, respectively, while at the same concentrations, ibuprofen inhibits this activity by 93.06% and 97.51%.

*(3) Effect of Methanolic Extract of D. benthamianus on the Inhibition of 5-Lipoxygenase Activity*. To study the effect of the extract on the production of leukotrienes, evaluation of the effect of the extract on the activity of 5-lipoxygenase was used. The results show that the methanolic extract of *D. benthamianus* and ibuprofen at the concentration of 1000 *μ*g/ml significantly inhibit (*p* < 0.001) the activity of 5-lipoxygenase with respective inhibition percentages of 81.54% and 95.65% (Table 1).

#### 4.1.2. Immunomodulatory Assay

*(1) Effect of Methanolic Extract of D. benthamianus on the Production of Intracellular and Extracellular ROS*. The effect of the methanolic extract of *D. benthamianus* on the production of reactive species of intracellular oxygen stimulated by zymosan and extracellular stimulated by PMA was evaluated (Table 2). For intracellular ROS, the methanolic extract of *D. benthamianus* shows significant inhibitory activity with an IC_50_ of 9.47 ± 0.12 *μ*g/ml in the blood; 5.59 ± 0.03 *μ*g/ml by PMN, and 6.05 ± 0.025 *μ*g/ml by macrophages. Concerning extracellular ROS, the methanolic extract of *D. benthamianus* inhibits their production with IC_50_ of 8.9 ± 0.0921 *μ*g/ml; 4.40 ± 0.10 *μ*g/ml; and 5.29 ± 0.37 *μ*g/ml, respectively, in the blood, by PMN, and by macrophages.

*(2) Effect of Methanolic Extract of D. benthamianus on Cell Proliferation*. The extract was tested for its ability to inhibit cell proliferation at concentrations of 2, 10, and 50 *μ*g/ml. After stimulation with phytohemagglutinin-L, the methanolic extract of *D. benthamianus* showed a significant antiproliferative property.  Table 1 shows that the extract has antiproliferative activity with an IC_50_ of 3.01 ± 0.42 *μ*g/ml. Prednisolone used as a reference product inhibited cell proliferation with an IC_50_ lower than 3.10 *μ*g/ml.

*(3) Effect of Methanolic Extract of D. benthamianus on 3T3 Cells*. Concerning the effect of the extract on the viability of 3T3 cells, the results showed that the methanolic extract of *D. benthamianus* is nontoxic with an IC_50_ of 32.01 ± 0.87 *μ*g/ml compared to cycloheximide, a cytotoxic reference drug with an IC_50_ of 0.10 ± 0.13 *μ*g/ml (Table 1).

**Figure 1:**
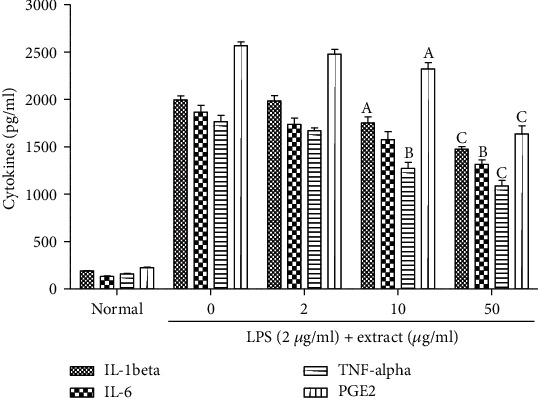
Effect of methanolic extract of *D. benthamianus* on proinflammatory cytokine production stimulated by LPS (lipopolysaccharides). Each value represents the mean ± SEM; ^a^*p* < 0.05, ^b^*p* < 0.01, and ^c^*p* < 0.001: significant difference compared to the normal group. The percentage values were obtained using various concentrations of test compounds, and readings are presented as mean of triplicates. IL: interleukin; TNF: tumor necrosis factor; PGE2: prostaglandin E2.

*(4) Effect of Methanolic Extract of D. benthamianus on the Production of Cytokines and PGE2*. The effect of the methanolic extract of *D. benthamianus* on the production of TNF-*α*, IL-1*β*, IL-6, and PGE2 by macrophages activated by LPS was evaluated (Figure 1). It was noted that at the concentration of 2 *μ*g/ml, the methanolic extract of *D. benthamianus* had no significant effect (*p* > 0.05) on the production of TNF-*α*, IL-1*β*, IL-6, and PGE2. At the concentration of 10 *μ*g/ml, the extract significantly inhibited the production of IL-1*β* (*p* < 0.05), TNF-*α* (*p* < 0.05), and PGE2 (*p* < 0.01). At the concentration of 50 *μ*g/ml, the extract significantly inhibited the production of TNF-*α* (*p* < 0.001), IL-1*β* (*p* < 0.001), IL-6 (*p* < 0.01), and PGE2 (*p* < 0.001).

### 4.2. *In Vivo* Assay

#### 4.2.1. Effect of Methanolic Extract of *D. benthamianus* on Chronic Ulcers

Five days after induction of gastric ulcers with ethanol, the animals had an index ulcer of 2.74. Four days after administration of indomethacin and without treatment, the index increases from 2.74 ± 0.02 to 3.00 ± 0.03. Treatment with methanolic extract of *D. benthamianus* produced a significant reduction (*p* < 0.001) in gastric lesions, with index values of 1.00 ± 0.00; 0.17 ± 0.16, and 0.00, corresponding to a healing percentage of 99.90%, 99.97%, and 100% in animals given 125, 250, and 500 mg/kg, respectively. An increase in mucus secretion of 83.17 ± 1.28 mg (125 mg/kg), 135.50 ± 4.26 mg (250 mg/kg), and 173.17 ± 3.56 mg (500 mg/kg) was also recorded in rats treated compared to the neutral control group (70.50 ± 4.24 mg), negative control 1 (48.50 ± 1.86 mg), and negative control 2 (32.00 ± 2.29 mg). The extract showed a higher activity than that developed by sucralfate (100 mg/kg; *p.o.*), which showed a healing of 36.22%, an ulcer index of 2.50 ± 0.01, and a weight mucus equal to 80.00 ± 1.69 mg (Table 3).

#### 4.2.2. Effect of Methanolic Extract of D*. benthamianus* on the Macroscopic of the Stomach

**Figure 2:**
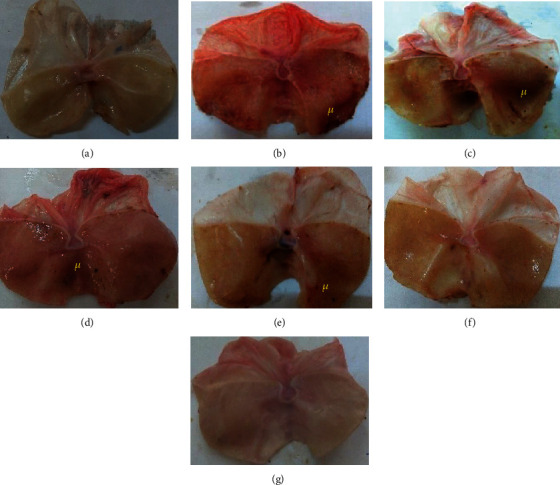
Macroscopic appearance of the gastric mucosa of the rats: (a) normal group: no lesions are observed; (b) ulcerated rats killed 5 days postethanol administration: severe lesions are seen in the gastric mucosa; (c) ulcerated rats (given indomethacin for 4 days + DMSO3% for 10 days): lesions are seen in the gastric mucosa with inflammation; (d) sucralfate (100 mg/kg): lesions in the gastric mucosa are less than those observed in negative control groups; (e) (125 mg/kg): less lesions; (f) (250 mg/kg); (g) (500 mg/kg): no lesions are observed; u: ulcer.

Oral administration of ethanol has resulted in gastric damage to the glandular part of the stomach (Figure 2). It appears from this figure that in normal rats, no lesion of the stomach wall is observed (Figure 2(a)). In the negative control 1 rats (Figure 2(b)) as in those of the negative control 2 (Figure 2(c)), inflammatory lesions were observed corresponding to the respective ulceration areas of 12.33% and 18.37%. Furthermore, the stomachs of animals treated with different doses of the methanolic extract of *D. benthamianus* show a significant reduction in inflammatory lesions corresponding to the ulcer surfaces of 0.02% (125 mg/kg, Figure 2(e)), 0.01% (250 mg/kg, Figure 2(f)), and 0.00% (500 mg/kg, Figure 2(g)).

#### 4.2.3. Effect of the Methanolic Extract of *D. benthamianus* on Some Hematological Parameters

Table 4 shows the effect of the extract on some immune cells 10 days after the administration of ethanol. It appears that the levels of white blood cells, monocytes, neutrophils, and eosinophils increase while the lymphocyte level decreases in animals of negative control groups 1 and 2 compared to the neutral control group. The treatment of rats with the different doses of methanolic extract of *D. benthamianus* leads to an improvement in the hematological parameters evaluated with a reduction in the levels of white blood cells, monocytes, neutrophils, and eosinophils and an increase in the lymphocyte level compared to animals from negative control groups 1 and 2.

#### 4.2.4. Effect of the Methanolic Extract of *D. benthamianus* on Some Tissue Parameters of Oxidative Stress

Some tissue parameters of oxidative stress were evaluated at the end of treatment with the different doses of the methanolic extract of *D. benthamianus*, and the results are presented in Table 5. It follows that the MDA level increases while the activities of catalase, SOD, and GSH decreased in rats in negative control groups 1 and 2 compared to animals in neutral control group. Treatment with methanolic extract of *D. benthamianus* and at all doses leads to a significant decrease in the level of MDA, then a significant increase in the activities of catalase, SOD, and GSH compared to animals of negative control groups 1 and 2.

#### 4.2.5. Effect of Methanolic Extract of *D. benthamianus* on the Histology of the Stomach Wall

**Figure 3:**
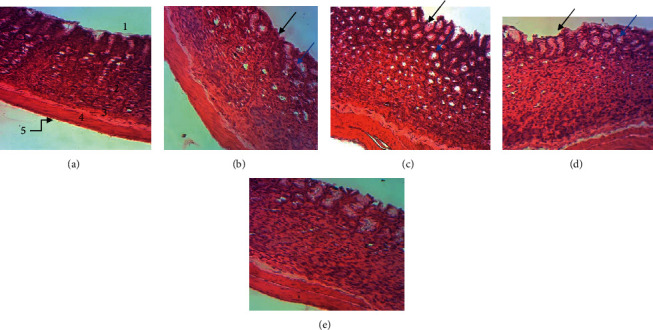
Histological study of ethanol-induced gastric damage in rats (H&E: ×400): (a) normal control rat: no injuries in the gastric mucosa are seen; (b) control 1 and (c) control 2: there is destruction of epithelium surface and edema; (d) sucralfate 100 mg/kg: the gastric wall appears with small destruction of epithelium and edema; (e) extract 500 mg/kg): there is complete cicatrization of ulcerated portion.

The stomach of rats which have not undergone ethanol induction of gastric ulcer shows a healthy gastric wall composed from the top to the bottom of the mucous membrane, the muscular mucosa, and the serosa (Figure 3(a)). The stomach of animals subjected to gastric ulcer induction without having received any treatment (Figures 3(b) and 3(c)) climbs a gastric wall with a lesion of the mucous layer (represented by the black arrow) and edemas (represented by the blue arrow). In animals treated with different doses of the methanolic extract of *D. benthamianus*, the stomachs show almost complete healing with significant reepithelialization and no edema (Figure 3(e)). Animals treated with sucralfate showed an incomplete but persistent process of reepithelialization of the ulcerated area (Figure 3(d)).

## 5. Discussion

In the present study, the results show that the methanolic extract of *D. benthamianus* has healing properties for chronic gastric ulcers induced by ethanol and/or indomethacin and via the anti-inflammatory and immunomodulatory mechanisms. Previous studies within the same project showed that the methanolic extract of *D. benthamianus* developed cytoprotective and antisecretory properties against acute ulcers in rats (published work). Additionally, the plant barks are widely used by Cameroonian populations as antiulcerative and on various gastrointestinal lesions of the mucous membranes. In the present study, we evaluated the healing effect of this plant, using an *in vivo* rat model of chronic gastric lesions induced by ethanol and indomethacin. Further, the mechanisms underlying this effect were evaluated using *in vitro* assays on the inhibition of protein denaturation, of COX and 5-LOX activities, of ROS production, of proinflammatory cytokines (TNF-*α*, IL-1*β*, IL-6) production, and of PGE2 and cell proliferation production. Our work has shown that the methanolic extract of *D. benthamianus in vivo* considerably reduces gastric lesions with a significant reduction in the ulceration index and a total recovery observed at a dose of 500 mg/kg, then in vitro inhibits significantly the denaturation of proteins, the activities of COX and 5-LOX, and the production of intracellular and extracellular ROS, proinflammatory cytokines, and PGE2 and also inhibits cell proliferation, exhibiting very low cytotoxicity.

One of the most important causes of the establishment and development of gastric ulcer in humans is ethanol, which is why gastric ulcer caused by the administration of ethanol in rats is a model essential for the preclinical study of new potentially antiulcer substances [31]. After administration of ethanol in rats, there are significant necrotic lesions and cell infiltration with significant reduction of defense factors (production of mucus, bicarbonate, and circulation of the mucous membranes) of the stomach wall [32–34]. However, it is known that one of the important elements in the protection of the stomach wall against multiple gastric lesions is the secretion of mucus [35]. In the present study, the methanolic extract of *D. benthamianus* significantly increased the secretion of mucus from the gastric wall, reduced the index of ulceration, and on histopathological sections confirmed the results by showing a normal mucous layer with a complete absence of gastric lesions in the treated groups compared to the untreated group. Furthermore, ethanol affects the gastric mucosa by destroying its barrier and causing microvascular changes; this leads to linear hemorrhagic lesions, mucosal friability, extensive submucosal edema, loss of epithelial cells in the stomach, and infiltration of inflammatory cells [36, 37]. These gastric lesions due to ethanol are thought to be the consequence of a direct or indirect action of ethanol on mediators such as cytokines, COX, 5-LOX, and free oxygen radicals [38]. In fact, after administration of ethanol, an inflammatory reaction sets in and is characterized by an increase in the proinflammatory cytokines (TNF-*α*, IL-1*β*, and IFN*γ*), the latter will stimulate significant cellular infiltration (neutrophils and macrophages), and then, TNF-*α* will restrict gastric microcirculation and thus delay healing [39–44]. In addition, it is known that the transcription factor NF-*κ*B, having the subunit NF-*κ*B-p65 as activation marker, plays an important role in gastric ulcer induced by ethanol as well as in the expression of various proinflammatory cytokines [40, 45]. Several authors have shown that ingestion of ethanol leads to an increase in oxidative stress and/or inflammatory cytokines which will phosphorylate the inhibitor (I*κ*B) of factor NF-*κ*B and increase the protein expression of NF-*κ*B p65 [44]. Our work has shown that the methanolic extract of *D. benthamianus* significantly inhibits the concentrations of TNF-*α*, IL-1*β*, IL-6, and PGE2. Thus, it is possible that the compounds present in this plant reduce the expression of the NF-*κ*B p65 subunit, reduce the phosphorylation of factor I*κ*B, and thus inhibit the activation of factor NF-*κ*B, leading to a significant decrease in the levels of TNF-*α* and IL-1*β*. This could be justified by the presence in this plant of terpenoids because several metabolites of this class exert their anti-inflammatory effect by inhibiting the phosphorylation of the factor NF-*κ*B [46, 47]; in addition, certain phenolic compounds such as gallic acid have shown their ability to interfere with various intracellular inflammatory pathways; in fact, it inhibits the expression of nuclear transcription factor NF-*κ*B and of the signal transducer, then regulates downwards their inflammatory targets [48].

In the model of induction of gastric lesions by NSAIDs such as indomethacin, the overproduction of proinflammatory cytokines (TNF-*α*, IL-1*β*, and IL-8) is considered to be an important inducer of these lesions [49]. NSAIDs cause the activation of the cell causing the phosphorylation of factor I*κ*B which is degraded followed by the release of NF-*κ*B, which is introduced into the nucleus of the cell and causes the transcription of numerous proinflammatory mediators including iNOS, COX-2, TNF-*α*, IL-1*β*, IL-6, and IL-8 [50]; thus, the aggravation of the inflammatory process in the pathophysiology of gastric ulcers would be due to an overproduction of cytokines and inflammatory mediators. Indeed, IL-1*β*, IL-8, and PGE2 just like TNF-*α* promote the inflammatory response and are strongly involved in the development and maintenance of gastric ulcers in humans [51–53]. Likewise, several authors have shown that in addition to cytokines capable of stimulating the production of free radicals and disturbing the microcirculation, the activation and/or the accumulation of neutrophils are also an important factor in lesions of the gastric wall due to NSAIDs [54]. Furthermore, according to the work of Anthony et al. [55], 15 to 30 min after administration of indomethacin in rats, there is a significant infiltration of neutrophils into the injured mucosa. Cellular infiltration is thus considered to be a crucial step in the development of lesions of the stomach wall caused by NSAIDs. The results of this study showed that the methanolic extract of *D. benthamianus* inhibits not only the production of proinflammatory cytokines (TNF-*α*, IL-1*β*, and IL-6) and the production of PGE2 but also cell proliferation. It is therefore possible that the secondary metabolites present in this plant exhibited a healing effect on gastric lesions through an anti-inflammatory action, by inhibiting the activities of COX and 5-LOX and by reducing the TNF-*α*, IL-1*β*, IL-6, and PGE2 secretions, thus leading to a reduction in mucosal cell proliferation with the consequence of stopping tissue destruction and reestablishing ulcers.

In rats, the administration of ethanol/indomethacin probably results in a significant contraction of the circular muscles of the fundic band, causing compression of the mucosa at the crests of the mucous folds, resulting in significant necrosis and ulceration as shown with ethanol by Mahmood et al. [56]. A molecule capable of causing the relaxation of these circular muscles can effectively protect the mucous membrane of the gastric wall by causing the flattening of the folds, which will have the advantage of increasing the areas of the mucous membrane exposed to necrotizing substances and thus reducing the quantity of these necrotizing substances on the crest of the stomach [57, 58]. This study showed on micrographs of flattening of the mucous folds, suggesting that the methanolic extract of *D. benthamianus* could exert its healing effect by a significant decrease in gastric motility. Because from the experimental point of view, Abdulla *et al*. [57] have shown that a decrease in gastric motility is an important element in the treatment of lesions of the gastric wall [59]. This is justified by the fact that Yousseu et al. [5] showed that the methanolic extract of *D. benthamianus* significantly reduced intestinal motility in rats; this activity is linked to the presence in this extract of gallic acid which exerts its effect by blocking calcium-dependent voltage channels and/or by inhibition of muscarinic receptors [6].

In the pathogenicity of inflammatory diseases, ROS are considered amplifiers of inflammatory proliferation; they play a key role insofar as their increase leads to an amplification of inflammation, activates or suppresses the transcription factor NF-*κ*B, induces the production of numerous cytokines, and activates enzymes such as COX and 5-LOX or even inducible nitrogen monoxide [60]. In many models of gastric lesions, tissue destruction dependent on the inflammatory process is the consequence of excessive recruitment and activation of neutrophils which will be responsible for the overproduction of free radicals [61]. Many cellular damage to our organism is the cause of ROS and free radicals which are generally produced continuously in our body; thus, the extracellular and/or intracellular antioxidants must continually protect the tissues against oxidative damage [62]. The gastric wall damage induced by ethanol/indomethacin is linked to an exaggerated breakdown of purine which leads to an overproduction of O_2_^−^ radicals and to an increase in lipid peroxidation mediated by ROS [63]. Lipid peroxidation is a very important pathophysiological event in various diseases, including gastric ulcer [64], because many mutagenic lesions are induced by the reaction between the MDA of lipid peroxidation and the bases of DNA [52]. Furthermore, it is known that in rats, the administration of antioxidants significantly reduces the gastric damage caused by ethanol and/or indomethacin [65]. The methanolic extract of D. benthamianus has shown significant antioxidant activity by reducing the level of MDA and increasing the activities of catalase, GSH, and SOD. Furthermore, this extract significantly inhibits the production of extracellular and intracellular ROS in whole blood and in various phagocytic cells (neutrophils and macrophages). This activity would be due to the inhibition of the production of proinflammatory cytokines (TNF-*α*, IL-1*β*, and IL-6), to the inhibition of protein denaturation, and to the inhibition of proinflammatory enzymatic activity, such as COX and 5-LOX. This can be justified by the fact that the gallic acid contained in the extract reduces the expression and/or activity of the proinflammatory cytokines and inflammatory proteins, including TNF-*α*, interferon-*γ* (INF-*γ*), IL-1*β*, IL-6, IL-17, IL-21, IL-23, cyclooxygenase (COX), and iNOS, and decreases expression and liberation of neutrophils and macrophages [48]. In addition, gallic acid improves the hepatotoxic effects of xenobiotic agents by acting as an antioxidant compound that eliminates free radicals, such as ROS, and improves the capacity of antioxidant defense systems [66].

Histological analysis of the stomach of the rats revealed the presence of lesions in the mucosa as well as edema in controls 1 and 2. This result shows an implication of the inflammatory process which took place in these controls. The normalization of the tissue in the rat treated with the methanolic extract of *D. benthamianus* causes reepithelialization of the mucosa, which shows that the extract would accelerate the healing of the ulcer and promote the regeneration of the gastric mucosa. The destruction of tissues and/or organs is very often the consequence of an unmodulated inflammatory response [67]. When there is a tissue disorder in the epithelium, a tissue repair program is immediately launched. The stomach crypts are an important reservoir of stem cells which first differentiate into progenitor cells and eventually become lineages of epithelial cells in order to activate the process. Thus, several previous studies have explained the role of TNF-*α* and IL-1*β* as indirect mediators of an endogenous tissue regeneration signal [68]. The layer of epithelial cells represents the second line of defense of the mucosa. This epithelial tissue is responsible for the production of mucus, bicarbonate, and other components of the mucobicarbonate barrier [69]. This result is in agreement with those obtained by Ateufack et al. [70] who have shown that the aqueous and methanolic extracts of *D. benthamianus* regenerate the gastric epithelium of rats subjected to acetic acid. In addition, the regenerative power of the extract has also been proven on the epithelium of two other organs, namely, the colon and the ileum by Yousseu et al. [5]. The compounds present in the extract would have activated several signaling pathways thus facilitating tissue reconstruction.

During the healing process of gastric ulcers, which is very complex and has several sequential phases (hemostasis, inflammation, proliferation, and remodeling), the tissues separate after the injury to restore the integrity of the mucosa [71]. It is known that controlling the production of stomach acid remains an important element in the healing of ulcers; however, the complex ulcer repair mechanisms show that the quality and speed of healing can be pharmacologically modulated. One of the main options being explored today is the use of dual COX and 5-LOX inhibitors which are able to prevent gastric mucosal ulcers from the exaggerated production of leukotrienes [72]. This is the case of licofelone which is a double inhibitor of COX and 5-LOX which can be administered for 4 to 12 weeks without altering the gastric mucosa in humans [73, 74]. Thus, with its antisecretory and cytoprotective properties, double inhibitor of COX/5-LOX, antioxidant properties, and inhibitors of the secretion of proinflammatory cytokines, the methanolic extract of *D. benthamianus* remains a good candidate for further studies in research of drugs which can bring about a complete cure of gastric ulcerations.

## 6. Conclusion

In conclusion, in vitro studies have shown that the methanolic extract of *D. benthamianus* has healing properties against gastric ulcers caused by ethanol and/or indomethacin. This effect would be linked to the inhibitory properties of the extract on protein denaturation; the activities of 5-LOX and COX; the production of ROS, proinflammatory cytokines, PGE2; and a decrease in the proliferation of lymphocytes. This effect can also be associated with the antioxidant properties and the ability to reepithelialize the plant. Thus, the presence of compounds such as gallic acid and other phenolic compounds may be partially responsible for these activities.

## Figures and Tables

**Table 1:** Effect of methanolic extract of *D. benthamianus* stem bark on protein denaturation, cyclooxygenase, and 5-lipoxygenase inhibition.

Treatment	Dose (*μ*g/ml)	Activity			Inhibition (%)		
		Protein denaturation	COX	5-LOX	Protein denaturation	COX	5-LOX
Control	—	0.518 ± 0.004	—	—	—	—	—

Diclofenac	100	0.133 ± 0.002^c^	—	—	74.41	—	—
	200	0.121 ± 0.002^c^	—	—	76.72	—	—
	500	0.133 ± 0.002^c^	—	—	81.48	—	—
	1000	0.056 ± 0.001^c^	—	—	89.19	—	—

Ibuprofen	100	—	0.11 ± 0.006	0.232 ± 0.022	—	83.51	83.16
	200	—	0.018 ± 0.001^c^	0.038 ± 0.001^c^	—	88.79	87.41
	500	—	0.011 ± 0.44^c^	0.029 ± 0.005^c^	—	93.06	91.36
	1000	—	0.007 ± 0.001^c^	0.021 ± 0.006^c^	—	97.51	95.65

Methanolic extract	100	0.215 ± 0.003^c^	0.003 ± 0.002	0.010 ± 0.001^c^	58.52	40.98	43.86
	200	0.205 ± 0.002^c^	0.045 ± 0.001^c^	0.080 ± 0.005^c^	60.39	56.15	59.42
	500	0.186 ± 0.002^c^	0.036 ± 0.003^c^	0.060 ± 0.004^c^	64.18	64.31	67.47
	1000	0.126 ± 0.006^c^	0.021 ± 0.001^c^	0.040 ± 0.003^c^	75.63	78.92	81.54

Each value represents the mean ± SEM; ^c^*p* < 0.001: significant difference compared to the control group. The percentage values were obtained using various concentrations of test compounds, and readings are presented as mean of triplicates. COX: cyclooxygenase; LOX: 5-lipoxygenase.

**Table 2:** IC_50_ value of methanolic extract of *D. benthamianus* stem bark on ROS production evaluated by zymosan/PMA-amplified chemiluminescence, on T-cell proliferation and Cytotoxicity.

Treatment	Intracellular and extracellular ROS						T cell proliferation (IC_50_ (*μ*g/ml))	Cytotoxicity (CI_50_ (*μ*g/ml))
	Luminol/zymosan (IC_50_ (*μ*g/ml))			PAM/lucigenin (IC_50_ (*μ*g/ml))				
	WB	PMNs	MQ	WB	PMNs	MQ		
Methanolic extract	9.47 ± 0.12	5.59 ± 0, 03	6.05 ± 0.025	8.91 ± 0.092	4.40 ± 010	5.29 ± 0.37	3.01 ± 0.42	32.01 ± 0.87
Ibuprofen	15.81 ± 0.22	15.20 ± 0.64	15.69 ± 1.45	17.83 ± 0.16	15.55 ± 0.54	16.57 ± 0.54	—	—
Prednisolone	—	—	—	—	—	—	<3.10	—
Cycloheximide	—	—	—	—	—	—	—	0.10 ± 0.13

The IC_50_ (median inhibitory concentration) values were obtained using various concentrations of test compounds, and readings are presented as mean ± standard deviation of triplicates. ROS: reactive oxygen species; WB: whole blood; PMNs: polymorphonuclear leukocytes; MQ: mice peritoneal macrophages.

**Table 3:** Effect of methanolic extract of *D. benthamianus* stem bark on ethanol-induced gastric lesions in rats.

Treatment	Dose (mg/kg)	UI	%US	% healing	Mucus weight (mg)
Neutral	/	0.00	0.00	/	70.50 ± 4.24
Control 1	/	2.74 ± 0.02	12.33	/	48.50 ± 1.86
Control 2	/	3.00 ± 0.03	18.37	/	32.00 ± 2.29
Sucralfate	100	2.50 ± 0.01	8.04	36.22	80.00 ± 1.69^cɣ^
Methanolic extract	125	1.00 ± 0.00^cɣ^	0.02	99.90	83.17 ± 1.28^cɣ^
	250	0.17 ± 0.16^cɣ^	0.01	99.97	135.50 ± 4.26^cɣ^
	500	0.00 ± 0.00^cɣ^	0.00	100	173.17 ± 3.56^cɣ^

Each value represents the mean ± standard error of the mean of 6 animals and analyses by one-way ANOVA followed by Tukey post hoc test; ^c^*p* < 0.001: significant when compared to negative control 1 (ulcerated rats killed 5-day postethanol administration); ^ɣ^*p* < 0.001: significant when compared to negative control 2 (received indomethacin for 4 days + 3% DMSO for 10 days). UI: ulcer index; US: ulcerated surface.

**Table 4:** Influence of the methanolic extract of *D. benthamianus* stem bark on some hematological parameters in ethanol-induced gastric lesions in rats.

Treatment	Dose (mg/kg)	TWBC (10^9^/l)	Lymphocyte (10^9^/l)	Monocytes (10^9^/l)	Neutrophil (10^9^/l)	Eosinophil (10^9^/l)
Neutral	/	18.00 ± 0.87	11.14 ± 0.60	1.12 ± 0.09	4.44 ± 0.27	1.30 ± 0.08
Control 1	/	23.15 ± 1.17	8.37 ± 0.31	2.22 ± 0.27	9.67 ± 0.41	3.02 ± 0.17
Control 2	/	20.17 ± 0.97	8.23 ± 0.28	2.13 ± 0.28	8.13 ± 0.55	3.83 ± 0.24
Sucralfate	100	16.50 ± 0.36^c*α*^	7.93 ± 0.21	1.17 ± 0.11^b*β*^	6.80 ± 0.38^c^	1.57 ± 0.08*^α^*
Methanolic extract	125	19.40 ± 0.96^a^	10.60 ± 0.06^b*β*^	1.07 ± 0.06^b*β*^	4.03 ± 0.18^cɣ^	2.97 ± 0.13
	250	17.92 ± 0.62^b^	8.87 ± 0.39	1.68 ± 0.21	5.73 ± 0.29^cɣ^	2.78 ± 1.22
	500	18.78 ± 0.47^a^	12.16 ± 0.50^cɣ^	1.46 ± 0.06	4.78 ± 0.09^cɣ^	1.44 ± 0.13*^α^*

Each value represents the mean ± standard error of the mean of 6 animals and analyses by one-way ANOVA followed by Tukey post hoc test; ^a^*p* < 0.05; ^c^*p* < 0.001: significant when compared to negative control 1 (ulcerated rats killed 5-day postethanol administration); *^α^p* < 0.05, ^ɣ^*p* < 0.001: significant when compared to negative control 2 (received indomethacin for 4 days + 3% DMSO for 10 days); TWBC: total white blood cells.

**Table 5:** Effect of methanolic extract of *D. benthamianus* stem bark on some parameters of oxidative stress in ethanol-induced gastric lesions in rats.

Treatment	Dose (mg∕kg)	MDA (*μ*mol/g of organ)	CAT (U/g of organ)	GSH (U/g of organ)	SOD (U/g of organ)
Neutral	/	2.07 ± 0.19	80.17 ± 0.02	7.14 ± 0.44	0.32 ± 0.02
Control 1	/	5.16 ± 0.02	34.21 ± 0.73	4.22 ± 0.08	0.27 ± 0.01
Control 2	/	5.10 ± 0.32	38.48 ± 0.33	4.96 ± 0.27	0.28 ± 0.01
Sucralfate	100	1.10 ± 0.00^cɣ^	53.49 ± 4.00	3.39 ± 0.03	0.29 ± 0.01
Methanolic extract	125	1.29 ± 0.12^cɣ^	66.31 ± 7.15^cɣ^	6.07 ± 0.37^cɣ^	0.22 ± 0.02
	250	1.38 ± 0.08^cɣ^	54.10 ± 2.13^cɣ^	7.09 ± 0.42^cɣ^	0.25 ± 0.01
	500	1.32 ± 0.03^cɣ^	47.87 ± 1.22^cɣ^	6.37 ± 0.32^cɣ^	0.24 ± 0.01

Each value represents the mean ± standard error of the mean of 6 animals and analyses by one-way ANOVA followed by Tukey post hoc test; ^c^*p* < 0.001: significant when compared to negative control 1 (ulcerated rats killed 5-day postethanol administration); ^ɣ^*p* < 0.001: significant when compared to negative control 2 (received indomethacin for 4 days + 3% DMSO for 10 days); MDA: malondialdehyde, CAT: catalase; GSH: glutathione; SOD: superoxide dismutase.

## Data Availability

All data supporting our findings are adequately contained within the manuscript.
